# Molecular identification of T-box transcription factor 6 and prognostic assessment in patients with congenital scoliosis: A single-center study

**DOI:** 10.3389/fmed.2022.941468

**Published:** 2022-08-11

**Authors:** Wenyan Zhang, Ziming Yao, Ruolan Guo, Haichong Li, Shuang Zhao, Wei Li, Xuejun Zhang, Chanjuan Hao

**Affiliations:** ^1^Beijing Key Laboratory for Genetics of Birth Defects, Beijing Pediatric Research Institute, Beijing, China; ^2^Ministry of Education of the People’s Republic of China (MOE) Key Laboratory of Major Diseases in Children, National Center for Children’s Health, Beijing Children’s Hospital, Capital Medical University, Beijing, China; ^3^Department of Orthopedics, National Center for Children’s Health, Beijing Children’s Hospital, Capital Medical University, Beijing, China; ^4^Henan Key Laboratory of Pediatric Inherited and Metabolic Diseases, Henan Children’s Hospital, Zhengzhou Hospital of Beijing Children’s Hospital, Zhengzhou, China

**Keywords:** congenital scoliosis (CS), T-box transcription factor 6 (*TBX6*), surgical treatment, prognosis, rare disease

## Abstract

**Background:**

Congenital scoliosis (CS) is characterized by vertebral malformations. The precise etiology of CS is not fully defined. A compound inheritance of *TBX6* was identified in 10% of patients with CS in Han Chinese and formed a distinguishable subtype named *TBX6*-associated congenital scoliosis (TACS).

**Methods:**

To investigate the variants and risk haplotype of *TBX6*, we recruited 121 patients with CS at Beijing Children’s Hospital. We collected the clinical characteristics and surgical treatment options and followed their postoperative prognoses.

**Results:**

Eight patients (6.6%) were molecularly diagnosed with TACS and carried the previously defined pathogenic *TBX6* compound heterozygous variants. All the eight patients with TACS had the typical TACS clinical feature of hemivertebrae in the lower part of the spine. These patients received posterior hemivertebra resection combined with segmental fusion. Follow-ups revealed satisfactory correction without postoperative complications.

**Conclusion:**

We observed a 6.6% prevalence of TACS in our CS cohort. Follow-ups further highlighted that surgical treatment of hemivertebra resection combined with segmental fusion performed well with prognosis for patients with TACS. This could provide valuable information for CS individuals with compound heterozygosity in *TBX6*.

## Introduction

With an incidence rate of 0.5–1/1,000 of live births, congenital scoliosis (CS) is the most frequent congenital deformity of the spine that causes birth defects (1,2). CS is caused by vertebral deformities including defects of formation or segmentation, or a combination of both. Patients present unbalanced body axis growth and curvature of the spine, and scoliosis progresses rapidly with the patient’s growth. Because of progressive scoliosis and secondary thoracic insufficiency caused by rib deformity, the patient quality of life is considerably compromised. Thus, most patients require more than one timely and effective treatment to correct scoliosis and avoid limitations of lung function (3).

Genetic studies have identified a series of genes, including Notch signaling pathway genes, genes encoding enzymes that regulate vertebral metabolism, ion channel genes, and ciliopathy-associated genes, associated with vertebral malformations and CS (4). Among these genes, T-box transcription factor 6 (*TBX6*), involved in activating the expression of HES family bHLH transcription factor 7 (*HES7*), mesoderm posterior basic helix-loop-helix transcription factor 2 (*MESP2*), and RIPPLY transcriptional repressor 2 (*RIPPLY2*) and regulating the cyclical activation of Notch signaling (5–10), has been identified as an essential regulator in the development of somites (11). Deleterious homozygote or compound heterozygote mutations in the *TBX6* gene could lead to spondylocostal dysostosis 5 [online mendelian inheritance in man (OMIM) 122600] (12–14) (Supplementary Table 1). Patients with *TBX6*-related spondylocostal dysostosis 5 present with rib deformities and a short trunk, which is caused by extensive vertebral deformities such as hemivertebrae, butterfly vertebrae, and vertebral fusion (12–14). A CS cohort study in Han Chinese showed that the compound inheritance of *TBX6* included a rare null mutation and a common risk haplotype T-C-A (composed of the three single-nucleotide polymorphisms, namely, rs2289292, rs3809624, and rs3809627) could lead to *TBX6*-associated congenital scoliosis (TACS), which accounted for 10% of patients with CS in this cohort (15). Patients with TACS presented with hemivertebrae in the lower half of the spine with or without mild rib deformities. The TACScore scoring system was developed to evaluate the likelihood of TACS (16). Subsequent studies in Japanese and European populations revealed similar inheritance patterns, population frequency, and clinical features of TACS (13,17). In contrast, the frequency of TACS in Hong Kong and Texas, United States was as low as 4.5% (18,19). However, published studies have mainly focused on adolescent patients (15,19), while those patients who present in childhood and receive effective surgical treatment are rarely studied. Moreover, the surgical treatment of patients with TACS has not been systematically reported and patient prognosis has not been followed.

In this study, we enrolled 121 patients with CS who visited Beijing Children’s Hospital for surgical treatment and genetic counseling. In the single-center cohort, eight (6.6%) patients were genetically diagnosed with TACS. We collected information on clinical manifestations and postoperative prognoses during follow-up. All the patients with TACS had one or more malformed vertebrae in the lower part of the spine and consented to posterior hemivertebra resection combined with segmental fusion surgery, which effectively corrected scoliosis in patients with TACS with a mean correction rate of 90.84% and no feedback about complications. This finding could provide valuable information to guide clinical genetic counseling and the identification of treatment options, and provide further assurance to patients with TACS and their families.

## Materials and methods

### Ethical compliance

The study was approved by the Institutional Medical Ethics Committee of Beijing Children’s Hospital, Capital Medical University (Approval No. 2015-26). All the patients or legal guardians provided written informed consent for this study.

### Subjects

We enrolled patients that were diagnosed with CS by at least two independent surgeons in the Department of Orthopedics of Beijing Children’s Hospital from January 2019 to December 2021. Patients were referred to the Orthopedics Department for surgical and genetic consults. A panel of trained physicians and geneticists at Beijing Children’s Hospital assessed patients for genomic testing. We collected peripheral blood samples of patients and their parents, documented surgical treatment, and followed prognoses until January 2022. Images, including CT, X-ray, and MRI, were collected before and after surgery to evaluate prognosis. The Cobb angle was measured using Surgimap version 2.3.2.1 software (Nemaris Incorporation, New York, United States). TACScore was calculated as previously described (16). The correction rate was calculated using the following equation.


Correction⁢rate=

$$\frac{\mathrm{Cobb}\,\mathrm{angle}\,\mathrm{before}\,\mathrm{surgery}-\mathrm{Cobb}\,\mathrm{angle}\,\mathrm{in}\,\mathrm{latest}\,\mathrm{follow}-\mathrm{up}}{\mathrm{Cobb}\,\mathrm{angle}\,\mathrm{before}\,\mathrm{surgery}}\times 100\%$$

### Exome sequencing

**FIGURE 1:**
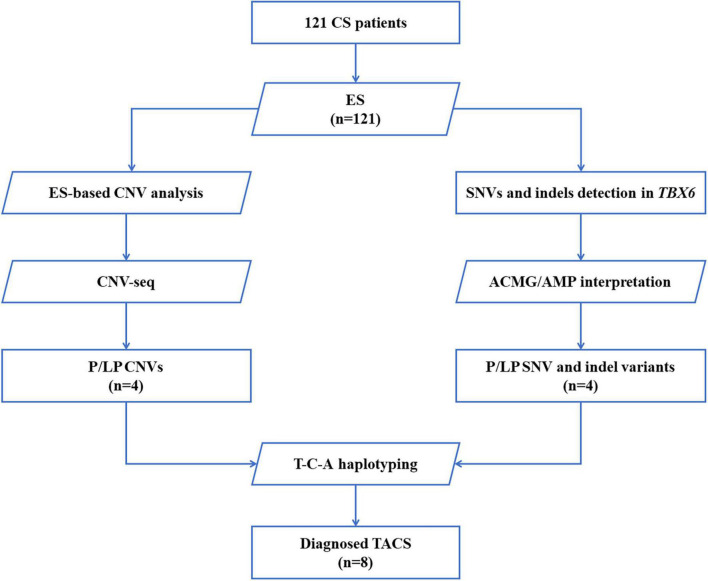
Flow diagram of patients with CS who were enrolled and received genetic testing. A total of 121 patients were recruited, and exome sequencing was performed. SNVs and indels in *TBX6* were evaluated by American College of Medical Genetics and Genomics (ACMG)/Association for Molecular Pathology (AMP) guidelines. CNVs were first analyzed based on ES data, further confirmed by CNV-seq, and classified by reported guidelines. The DNA of patients with pathogenic/likely pathogenic *TBX6* variants (right branch) or *TBX6*-involving CNVs (left branch) was amplified and sequenced to verify their T-C-A haplotypes. CS, congenital scoliosis; ES, exome sequencing; SNV, single nucleotide variation; indel, inversion and deletion; CNVs, copy number variations; ACMG/AMP interpretation, American College of Medical Genetics and Genomics and the Association for Molecular Pathology interpretation standards and guidelines; P, pathogenic; LP, likely pathogenic; T-C-A, rs2289292 (C > T) - rs3809624 (T > C) - rs3809627 (C > A); TACS, *TBX6*-associated congenital scoliosis.

Deoxyribonucleic acid was isolated from peripheral blood samples obtained from probands and their parents using the Gentra Puregene Blood Kit (Qiagen, Hilden, Germany). A total of 200 ng genomic DNA from each individual was sheared using Biorupter (Diagenode, Liège, Belgium) to acquire 150–200 bp fragments. The ends of the DNA fragment were repaired and Illumina Adaptors (Agilent Technologies, Santa Clara, United States) were added. After the sequencing library was constructed, the whole exome was captured using the SureSelect Human All Exon Kit (Agilent Technologies, Santa Clara, United States) and sequenced on Illumina NovaSeq 6000 (Illumina, San Diego, United States) with 150 base paired-end reads. Raw reads were filtered to remove low-quality reads using FastQC. Exome sequencing (ES) resulted in over 12 Gb of clean data. The average sequencing depth was greater than 100 X. Clean reads were mapped to the reference genome sequence Genome Reference Consortium Human Build 37 (GRCh37)/hg19 using Burrows–Wheeler Aligner and bam files were created using Picard. The Genome Analysis Toolkit software was used to perform variant calling (Figure 1).

### Variant analysis and copy number variation calling based on exome sequencing data

Single-nucleotide variants (SNVs) were annotated and filtered using TGex.^1^ Variants with a frequency of over 1% in the Genome Aggregation Database (gnomAD), NHLBI Exome Sequencing Project (ESP), or 1,000 G databases were excluded. Variants that lacked segregation in family members were also filtered. The main disease reference databases were Human Gene Mutation Database (HGMD) Professional, ClinVar, OMIM, and MalaCards. The pathogenicity of the missense variants found in patients was evaluated using the following bioinformatics tools: PolyPhen-2 (version 2.2.2),^2^ protein variation effect analyzer (PROVEAN) (version 1.03),^3^ MutationTaster,^4^ and VarCards^5^ (20). Variants were classified following the interpretation standards and guidelines of the American College of Medical Genetics and Genomics and the Association for Molecular Pathology (21). Putative pathogenic variants detected by next-generation sequencing were confirmed by Sanger sequencing. eXome-Hidden Markov Model (XHMM) software was used to detect copy number variations (CNVs) based on ES data (22) (Figure 1).

### Copy number variation sequencing

To confirm CNVs, part of the library without capture was sequenced directly onto Illumina NovaSeq 6000; each sample yielded 1 Gb of raw data (QC: average read length: > 0.3 × Whole-genome sequencing). An in-house pipeline was applied to map and call CNVs based on CNV sequencing software. Clean reads were mapped to the reference genome GRCh37/hg19. CNVs called from parental low-depth whole-genome sequencing data were used as controls. The following were excluded: (1) CNVs reported in multiple peer-reviewed publications as benign or likely benign; (2) CNVs annotated in curated databases as benign or likely benign; (3) CNVs observed frequently in the general population; and (4) CNVs that did not cover the coding regions of genes. The Database of Genomic Variants (DGVs), the DECIPHER database, ClinVar, OMIM, and ClinGen were used for the interpretation and classification of the clinical significance of candidate CNVs according to previously reported guidelines (23,24) (Figure 1).

### T-box transcription factor 6 T-C-A haplotype sequencing

Previously isolated DNA from patients and their parents was used for T-C-A haplotype sequencing, a technique first defined by Wu et al. (15). Among the three common single nucleotide polymorphisms (SNPs), C (rs3809624) and A (rs3809627) are located at the 5’ untranslated region and are close to one another (i.e., at a distance of 358 nt). In contrast, T (rs2289292) is located at the eighth exon of *TBX6*. Thus, we designed the amplifying and Sanger sequencing primers for C-A and T, respectively (Supplementary Table 2 and Figure 1).

### Statistical analysis

The Cobb angles were measured before and after surgery. Correction rates of patients were analyzed using SPSS version 20.0 software (IBM Incorporation, New York, United States). Graphs were drawn using Microsoft Excel (Microsoft Corporation, Washington, United States) and GraphPad Prism 8 (GraphPad Software, San Diego, United States).

## Results

### Demographics and clinical characteristics

**FIGURE 2:**
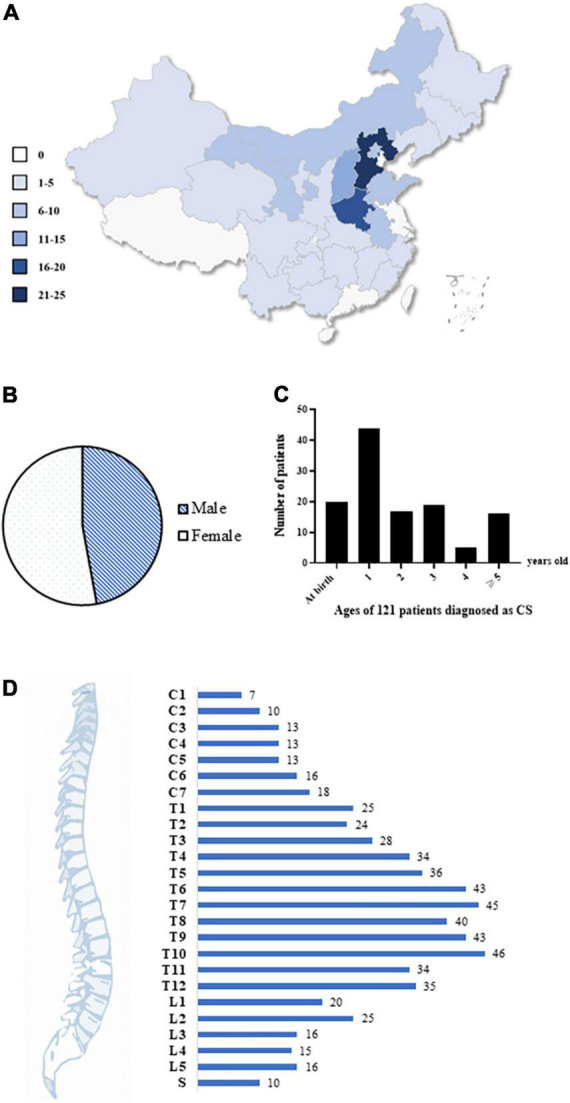
Demographics and clinical characteristics of the cohort. **(A)** The geographical characteristic of our cohort. A total of 121 Chinese patients with CS were recruited from all over the country. Different colors represent different patient numbers from the corresponding region, and the darker color, the more patients. **(B)** The gender ratio. A total of 57 males and 64 females were enrolled. **(C)** Age of 121 patients when they were diagnosed as CS. 87% of patients were diagnosed with CS before 5 years old. **(D)** The distribution of malformed vertebrae in all the patients. The numbers of patients were labeled. CS, congenital scoliosis; C, cervical; T, thoracic; L, lumbar; S, sacral.

We enrolled a total of 121 Chinese patients with CS nationwide (male:female = 57:64) (Figures 2A,B). Over 86% of patients were diagnosed with CS before the age of 5 years (Figure 2C). The spinal deformities of 121 patients originated from a vertebral formation defect and involved the entire spine, although T6–T10 were mostly affected (Figure 2D).

### Exome sequencing findings of T-box transcription factor 6

Exome sequencing was applied to all the patients and revealed one missense variant (Patient 1: c.745G > A/p.Val249Met), one nonsense variant (Patient 8: c.903T > G/p.Tyr301*), and two frameshift variants (Patient 6: c.271dupG/p.Val91fs × 79; Patient 7: c.1115_1130dupAGGCTCCAGACTCCGG/p.Arg378fs × 66) of *TBX6* (Table 1). All the four variants were not recorded in the gnomAD, ExAC, or 1,000 G databases, indicating that the population frequencies of these variants were extremely low. *In silico* analyses indicated that the unreported *TBX6* missense variant from patient 1 was deleterious (Supplementary Table 3). Thus, all the four variants were pathogenic mimicking a 16p11.2 microdeletion.

**TABLE 1:** The genotypes of patients.

Patient	Allele 1	Allele 2
	**Deleterious variant**	**Common haplotype**
Patient 1	c.745G > A/p.Val249Met	T-C-A
Patient 2	16p11.2 deletion 0.54 Mb (chr16:29660000–30200000)	T-C-A
Patient 3	16p11.2 deletion 0.56 Mb (chr16:29640000–30200000)	T-C-A
Patient 4	16p11.2 deletion 0.52 Mb (chr16:29680000-30200000)	T-C-A
Patient 5	16p11.2 deletion 0.56 Mb (chr16:29640000–30200000)	T-C-A
Patient 6	c.271dupG/p.Val91fs*79	T-C-A
Patient 7	c.1115_1130dupAGGCTCCAGACTCCGG/p.Arg378fs*66	T-C-A
Patient 8	c.903T > G/p.Tyr301*	T-C-A

The haplotype defined by three common TBX6 SNPs (reference/non-reference): rs2289292 (C/T)–rs3809624 (T/C)–rs3809627 (C/A).

The “*” was one of the genetic terms. In “c.271dupG/p.Val91fs*79” it means frameshift occurs at the 91st amino acid, translation is stopped after 79 amino acids. So does “c.1115_1130dupAGGCTCCAGACTCCGG/p.Arg378fs*66”. In “c.903T > G/p.Tyr301*”, it means the 301st amino acid turns into a termination codon and translation is stopped.

### Copy number variation analysis of T-box transcription factor 6

Copy number variation calling based on ES data suggested that four of the 121 patients with CS (patients 2–5) had a heterozygous deletion of the 16p11.2 region. Further CNV sequencing tests confirmed the deletions. Patients 2–5 consistently carried 0.52–0.56 Mb of heterozygous deletions of *TBX6* and all the deleted regions covered the entire *TBX6* gene (Table 1 and Supplementary Figure 1).

### Haplotyping of T-box transcription factor 6 variants

**FIGURE 3:**
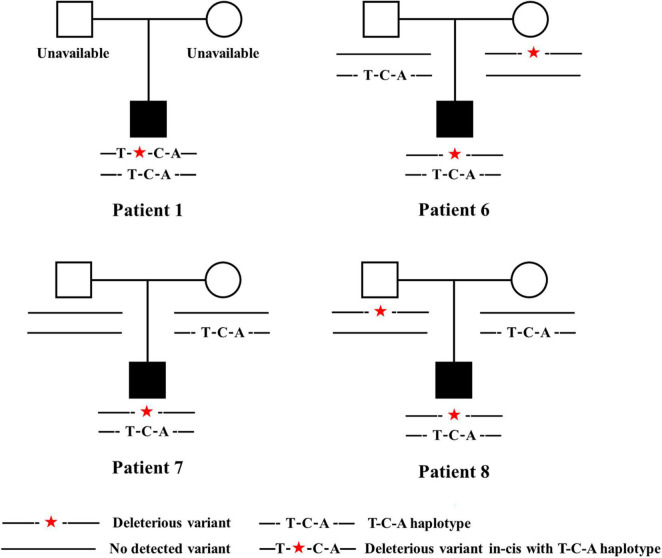
Pedigrees of patients carrying *TBX6* deleterious variants in-trans with T-C-A haplotypes. Patient 1 had c.745G > A/p.Val249Met, Patient 6 had c.271dupG/p.Val91fs × 79, Patient 7 had c.1115_1130dupAGGCTCCAGACTCCGG/p.Arg378fs × 66, and patient 8 had c.903T > G/p.Tyr301*. Legends are listed below. T-C-A, rs2289292 (C > T) – rs3809624 (T > C) – rs3809627 (C > A).

To determine whether the T-C-A haplotype existed in patients, T and C-A were amplified and PCR productions were sequenced separately. All the eight patients carried the T-C-A haplotype with in-trans *TBX6* deleterious variants, representing typical TACS compound inheritance (Table 1 and Figure 3).

### Clinical characteristics of patients with T-box transcription factor 6-associated congenital scoliosis

**FIGURE 4:**
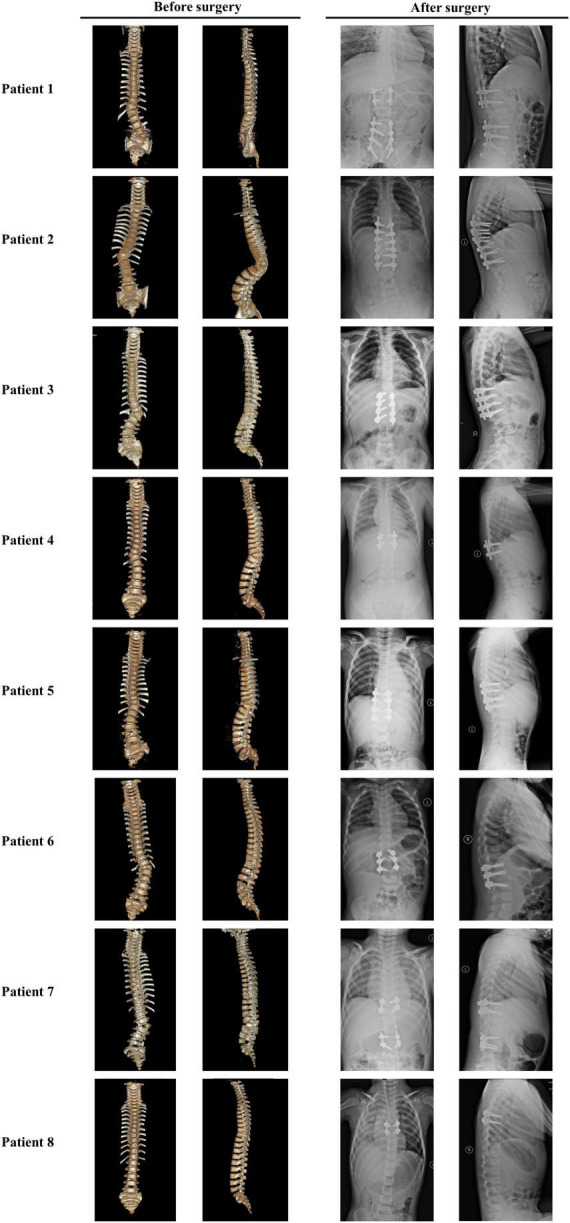
Images of patients with TACS before and after surgeries suggested well outcomes. **Patient 1** had hemivertebrae at T12 and L5 with the Cobb angle of 26°; after surgery, the Cobb angle was reduced to 2°. **Patient 2** had hemivertebrae at T11 with the Cobb angle of 53°; after surgery, the Cobb angle was reduced to 87°. **Patient 3** had hemivertebrae at T12 and butterfly vertebrae at T11 with the Cobb angle of 50°; after surgery, the Cobb angle was reduced to 5°. **Patient 4** had hemivertebrae at T11 with the Cobb angle of 41°; after surgery, the Cobb angle was reduced to 0°. **Patient 5** had hemivertebrae at T11 and butterfly vertebrae at L4 and S3 with the Cobb angle of 30°; after surgery, the Cobb angle was reduced to 3°. **Patient 6** had hemivertebrae at T12 and butterfly vertebrae at L5 with the Cobb angle of 46°; after surgery, the Cobb angle was reduced to 6°. **Patient 7** had unsegmented hemivertebrae at T10 and L3 and butterfly vertebrae at C7 and T9 with the Cobb angle of 22°; after surgery, the Cobb angle was reduced to 3°. **Patient 8** had hemivertebrae at T8 with the Cobb angle of 26°; after surgery, the Cobb angle was reduced to 4°. Images before and after surgeries were obtained by CT and X-ray, respectively. TACS, *TBX6*-associated congenital scoliosis.

Eight patients (five boys and three girls) were diagnosed with TACS, which accounted for 6.6% of our CS cohort. All the patients had malformed vertebrae located in the lower half of the spine (below T8) and only patient 7 had an additional butterfly vertebra at C7 (Figure 4). Patients 2, 3, and 8 had mildly deformed ribs. In addition, patient 5 exhibited mild developmental delay and patient 8 had bilateral radial polydactyly. In summary, these patients displayed the typical clinical characteristics of TACS despite only four patients scoring ≥ 3, the cutoff point, according to the TACScore algorithm (Table 2).

**TABLE 2:** Clinical information of patients.

	Patient 1	Patient 2	Patient 3	Patient 4	Patient 5	Patient 6	Patient 7	Patient 8
**Gender**	Male	Female	Female	Female	Male	Male	Male	Male
**Delivery**	Uneventful	Uneventful	Uneventful	Uneventful	Cesarean section because of macrosomia	Uneventful	Uneventful	Uneventful
**Age of finding scoliosis**	1 year	1 year	5 month	2 month	1 year	1 year	1 year	Intrauterine 7 month
**Age of undergoing surgeries**	3 year	2, 8 year	8 year	4 year	6 year 4 month, 6 year 7 month	2 year	2, 5 year	4 year 2 month
**Hemivertebra**	T12, L5	T11	T12	T11	T11	T12	T10, L3 (unsegmented)	T8
**Butterfly-vertebra**	−	−	T11	−	L4, S3	L5	C7, T9	−
**Fusional vertebra**	−	T7–L2	−	−	−	−	T9–10, L3–4	−
**Rib malformation**	−	Yes	Yes	−	−	−	−	Yes
**Other clinical features**	−	Kyphosis	−	−	Mildly developmental delay	−	−	Polydactyly at bilateral thumb radial
**TACScore**	3	2	4	3	2	3	2	3
**Cobb angel before surgery (°)**	26	53	50	41	30	46	22	26
**Number of operations**	2	1	1	1	2	1	2	1
**Cobb angel after last surgery (°)**	2	6	5	0	3	6	3	4
**Correction rate (%)**	93.8	88.7	90.0	100.0	90.0	87.0	86.4	84.6
**Fusion occurs at last follow-up**	Yes	Yes	Yes	Yes	Yes	Yes	Yes	Yes
**Complication**	No	No	No	No	No	No	No	No
**Follow-up time after surgery**	3 year 3 month	3 year	3 year	3 year	2 year 9 month	1 year	2 year 3 month	3 month
**Surgery**	Posterior hemivertebra resection and segmental fusion	Posterior hemivertebra resection and segmental fusion	Posterior hemivertebra resection and segmental fusion	Posterior hemivertebra resection and segmental fusion	Posterior hemivertebra resection and segmental fusion^†^	Posterior hemivertebra resection and segmental fusion	Posterior hemivertebra resection and segmental fusion^†^	Posterior hemivertebra resection and segmental fusion

Y, year; M, month; C, cervical; T, thoracic; L, lumbar; S, sacral; TACScore, TBX6-associated congenital scoliosis score.

^†^Patient 5 and 7 underwent twice surgeries of posterior hemivertebra resection and segmental fusion.

### Surgical strategy and patient prognosis

All the patients with TACS were diagnosed with scoliosis before the age of 1 year and underwent surgery at varying ages. Because of the presence of simple hemivertebrae, all the patients received corresponding hemivertebra resection combined with short segmental fusion. Patients 1, 5, and 7 underwent surgery twice for two malformed vertebrae each, which progressed as patients grew. Before surgery, patients’ Cobb angles ranged from 22.0 to 53.0° with a mean angle of 38.28 ± 12.28°. In the most recent follow-up (i.e., at 3 years and 3 months), the Cobb angles ranged from 0 to 6.0° and the mean angle was 3.57 ± 2.22°. Correction rates ranged from 86.4 to 100% with a mean rate of 90.84 ± 4.71% and no complications were reported (Table 2 and Figure 4).

## Discussion

In this study, we enrolled 121 patients with CS who visited Beijing Children’s Hospital for surgical treatment and genetic counseling; eight of these patients were molecularly diagnosed with TACS. Patients with TACS accounted for 6.6% of CS. Our study retrospectively summarized the clinical characteristics of patients with TACS and the surgical strategies adopted for these patients. The study is the first study to report the postsurgical prognosis of patients with TACS and suggests that all the patients had positive outcomes.

The 6.6% prevalence of TACS among patients with CS was slightly different from that reported in the following CS cohorts of previous studies: 10% in Han Chinese (15), 9.6% in a Japanese cohort (17), 7.2% in a European cohort (13), and 4.5% in a Hong Kong, Southern China cohort (19). Several explanations for this difference are proposed. First, ethnic differences in these regional populations likely contribute to different allele frequencies. Second, with the development of prenatal ultrasound diagnostic technology, fetuses with CS accompanied by severe structural malformations can be identified and pregnancy terminated. Third, the TACS ratio may have been altered with the enlargement of our cohort.

Previous Deciphering Disorders Involving Scoliosis and Comorbidities (DISCO) studies have revealed that the TACScore is a cost-effective and time-saving tool for screening patients with TACS and shows considerable sensitivity (93.9%), specificity (90.9%), and accuracy (91.2%). However, the sensitivity, specificity, and accuracy of the TACScore were 50, 85.8, and 83.5%, respectively, in our cohort, far lower than the values in DISCO studies. We inferred that results could be due to our smaller cohort size and that findings may more closely match those in previous reports if our cohort is expanded (16, 25).

In clinical practice, the selection of surgical treatment comprehensively considers three indicators: patient age, the Cobb angle magnitude, and the type of malformation. These indicators predict the surgical correction rate and operative and postoperative complications. For hemivertebrae-induced CS, posterior hemivertebra resection combined with segmental fusion was the most common choice of surgery. Its correction rate varied from 46 to 87% (26–35). The frequency of complications ranged from 0 to 41% (27,28,32,33,35–42) and complications included wound infection, adding-on phenomenon, pseudoarticulation formation, postoperative progression of scoliosis, the offset of the internal fixation, and pedicle fractures. In our previous study (43), it showed that 14 patients with CS younger than 5 years of age accepted short fixation for posterior hemivertebra resection, which had a mean correction rate of 77.86% and no surgery-related complications. With a mean correction rate of 90.84%, posterior hemivertebra resection combined with segmental fusion was more effective in patients with TACS. Taken together, our findings implied that the optimal surgical choice for patients with TACS is posterior hemivertebra resection combined with segmental fusion, which should be a first treatment option for patients with TACS.

As a complex genetic disorder, CS is often accompanied by multisystem abnormalities. For example, patient 5 with 16p11.2 deletion presented with mild developmental delay and intellectual disability. The 16p11.2 deletion is considered to be related to autism, obesity, developmental delay, and intellectual disability (13,44–46). Comprehensive and systematic physical examination is required for clinical diagnosis and treatment and CS may present a considerable psychological and financial burden to children and their families (3). Although the clinical phenotypes of patients with TACS are less complicated than those of other malformations, most orthopedic clinicians find TACS difficult to distinguish from other forms of non-syndromic scoliosis. Genetic testing for *TBX6* can rapidly identify the etiology and reduce unnecessary physical examination (15). In addition, CS caused by different gene mutations exhibits varying patterns of progression. A definite molecular diagnosis can further predict complications and long-term prognosis and can guide the identification of appropriate intervention time and surgical options (47). In our study, all the molecularly diagnosed patients with TACS received posterior hemivertebra resection combined with short-stage fusion and the prognoses were followed for at least 1 year. All the patients ultimately presented with the Cobb angle of less than 10°, an average correction rate of 90.84%, and no complications to date. Thus, our results indicated that hemivertebrae caused by the compound inheritance of *TBX6* can be corrected effectively by hemivertebra resection combined with segmental fusion—a finding that may reassure CS children and their parents.

## Conclusion

In conclusion, we recruited 121 patients with CS in Beijing Children’s Hospital and diagnosed eight (6.6%) of these patients with TACS with *TBX6* deleterious and hypomorphic allele in trans. Follow-up revealed that hemivertebra resection and segmental fusion resulted in positive outcomes. Our study can guide risk evaluation for patients with TACS and their families.

## Data availability statement

The data that support the findings of this study are openly available. Raw data were uploaded and public to gsa-human [Genome Sequence Archive for Human (https://ngdc.cncb.ac.cn/gsa-human/)], under accession PRJCA010512.

## Ethics statement

The studies involving human participants were reviewed and approved by the Institutional Medical Ethics Committee of Beijing Children’s Hospital, Capital Medical University (BCH, Approval No. 2015-26). Written informed consent to participate in this study was provided by the participants’ legal guardian/next of kin.

## Author contributions

CH and XZ conceived and designed the study. ZY, HL, and XZ recruited their respective patients for this study and provided clinical data. WZ conducted the experiments, analyzed the data, and wrote the manuscript. RG and SZ contributed to the analysis of sequencing data. CH and WL were involved in manuscript editing. All authors reviewed and approved the final version of the manuscript.

## Footnotes

1
https://fa.shanyint.com

2
http://genetics.bwh.harvard.edu/pph2/

3
http://provean.jcvi.org/

4
http://www.mutationtaster.org/

5
http://varcards.biols.ac.cn/
