# Supplementary material for: Molecular identification of T-box transcription factor 6 and prognostic assessment in patients with congenital scoliosis: A single-center study
Source: Front Med (Lausanne). 2022 Aug 11;9:941468. doi: 10.3389/fmed.2022.941468 (PMC9403053; doi:10.3389/fmed.2022.941468)
Supplement: Supplementary file 1 [file Data_Sheet_1.PDF]

Supplementary Table 1. Reported *TBX6* variants leading to SCD

Case	Variant	Effect of Variant	Clinical Feature
Sparrow et al. [1]	Heterozygous c.1013C>T (p.*437Cext*81)	Extended 81 amino acids affected interaction between TBX6 and other proteins. The transcriptional activating activity is significantly reduced.	All of three patients present VSD throughout spine, including hemivertebrae and fused vertebral blocks leading to scoliosis and short trunk.
Lefebvre et al. patient 3 [2]	16p11.2 deletion<br>c.661C>A (p.H221N)	Null of TBX6.<br>Mis-localization of TBX6 protein.	Extended VSD with a “pebble beach” appearance and hemivertebrae.<br>Malformed ribs.
Lefebvre et al. patient 4 [2]	c.699G>C (p.W233C)<br>c.422T>C (p.L141P)	Mis-localization of TBX6 protein.<br>Reduced transcriptional activity.	Multiple VSD affecting more than 10 continuous levels, wedge-shaped vertebrae and hemivertebrae.<br>Malformed ribs.
Otomo et al. [3]	c.356G>A (p.R119H)<br>c.449G>A (p.R150H)	Mis-localization of TBX6 protein.<br>Mis-localization of TBX6 protein.	VSD from cervical to lumber spine.<br>Malformed ribs.
Chen et al. patient US-P2[4]	Homozygous c.418C>T (p.L140F)	Mis-localization of TBX6 protein and reduced transcriptional activity.	VSD from thoracic to lumbar spine.<br>Multilevel rib, costovertebral joint ankylosis.

TBX6, T-box transcription factor 6; SCD, spondylocostal dysostosis; VSD, vertebral segmental defect.

Supplementary Table 2. Primers designed for T-C-A amplifying and sequencing

Target	Primer
T (rs2289292)	Forward: 5’-GGGTGGGAGTGAAATCAA-3’<br>Reverse: 5’-CAGGTTATGTCGGACAGTAAA-3’
C-A (rs3809624, rs3809627)	Forward: 5’-CGGGGAAGAATGAGGAGC-3’<br>Reverse: 5’-GCCTGCCGGGAAGTGTAG-3’

Supplementary Table 3. The pathogenicity evaluation of *TBX6* missense variant in Patient 1

				Allele frequency				*In-silico* tool					
Patient	Nucleotide change	Amino acid change	Opposite haplotype	ExAC_all	gnomAD_all	1000 Genomes	dbSNP	PolyPhen-2	Mutation taster	PROVEAN	SIFT	VarCards	Classification
1	c.745G>A	p.Val249Met	T-C-A	0	0	0	0	1 (PD)	1 (D)	-2.92 (D)	0	Y	LP

T-C-A, rs2289292 (C>T) - rs3809624 (T>C) - rs3809627 (C>A); ExAC_all, the Exome Aggregation Consortium, all population; GnomAD_all, the Genome Aggregation Database, all population (http://gnomad.broadinstitute.org/); 1000 Genomes, The 1000 Genomes Project (https://www.internationalgenome.org/); dbSNP, database of single nucleotide Polymorphism (https://www.ncbi.nlm.nih.gov/snp/);PD, Probably damaging of PolyPhen-2 (v2.2.2; http://genetics.bwh.harvard.edu/pph2/); D, Disease causing of PROVEAN (v1.03;http:// http://provean.jcvi.org/); D, deleterious of MutationTaster (http://www.mutationtaster.org/); 0, SIFT score of SIFT (http://sift.bii.a-star.edu.sg/); Y, extreme variant of VarCards (http://varcards.biols.ac.cn/).

Patient 2

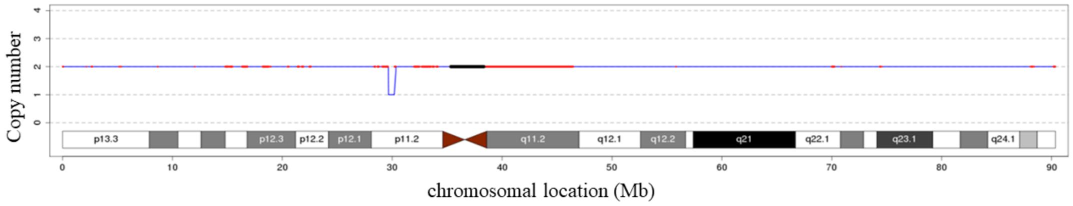

Patient 3

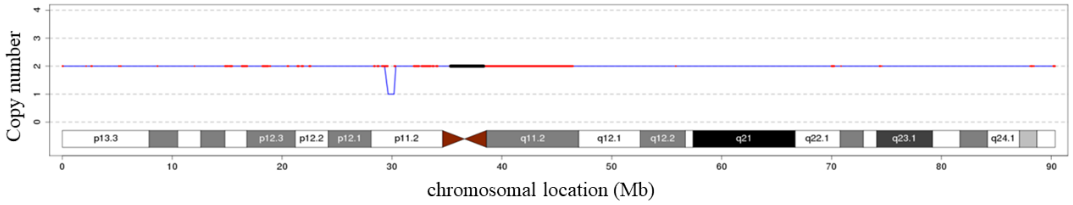

Patient 4

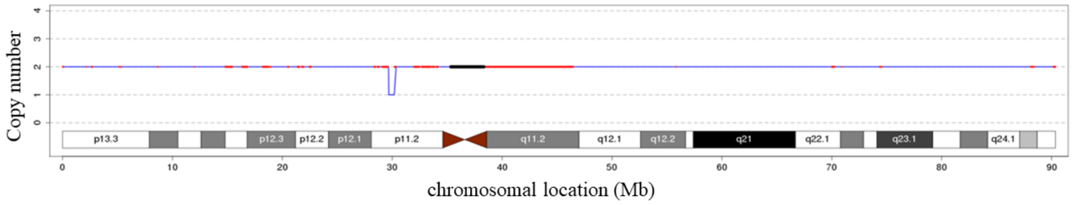

Patient 5

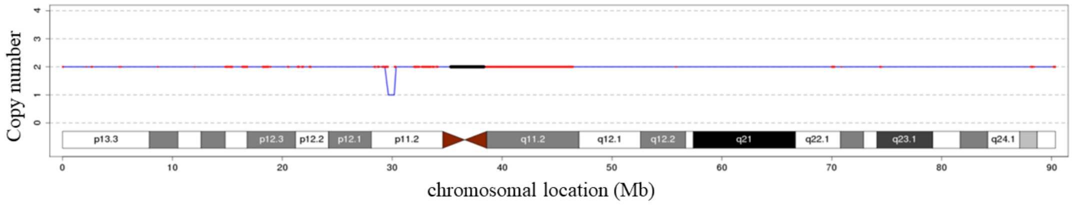

Supplementary Figure 1. CNV-seq results revealed 16p11.2 deletions of Patient 2-5. Deleted regions of Patient 2-5 were 29660000-30200000 (0.54Mb), 29640000-30200000 (0.56Mb), 29680000-30200000 (0.52Mb), and 29640000-30200000 (0.56Mb) of chromosome 16 respectively, which included *TBX6* gene.
